# Perceptual Color Space Representations in the Oculomotor System Are Modulated by Surround Suppression and Biased Selection

**DOI:** 10.3389/fnsys.2018.00001

**Published:** 2018-01-26

**Authors:** Devin H. Kehoe, Maryam Rahimi, Mazyar Fallah

**Affiliations:** ^1^Department of Psychology, York University, Toronto, ON, Canada; ^2^Centre for Vision Research, York University, Toronto, ON, Canada; ^3^Vision Science to Applications (VISTA), York University, Toronto, ON, Canada; ^4^Canadian Action and Perception Network, York University, Toronto, ON, Canada; ^5^School of Kinesiology and Heath Science, York University, Toronto, ON, Canada

**Keywords:** oculomotor processing, oculomotor representations, color, memory-guided saccades, saccade curvature, target selection, surround suppression, color hierarchy

## Abstract

The oculomotor system utilizes color extensively for planning saccades. Therefore, we examined how the oculomotor system actually encodes color and several factors that modulate these representations: attention-based surround suppression and inherent biases in selecting and encoding color categories. We measured saccade trajectories while human participants performed a memory-guided saccade task with color targets and distractors and examined whether oculomotor target selection processing was functionally related to the CIE (*x*,*y*) color space distances between color stimuli and whether there were hierarchical differences between color categories in the strength and speed of encoding potential saccade goals. We observed that saccade planning was modulated by the CIE (*x*,*y*) distances between stimuli thus demonstrating that color is encoded in perceptual color space by the oculomotor system. Furthermore, these representations were modulated by (1) cueing attention to a particular color thereby eliciting surround suppression in oculomotor color space and (2) inherent selection and encoding biases based on color category independent of cueing and perceptual discriminability. Since surround suppression emerges from recurrent feedback attenuation of sensory projections, observing oculomotor surround suppression suggested that oculomotor encoding of behavioral relevance results from integrating sensory and cognitive signals that are pre-attenuated based on task demands and that the oculomotor system therefore does not functionally contribute to this process. Second, although perceptual discriminability did partially account for oculomotor processing differences between color categories, we also observed preferential processing of the red color category across various behavioral metrics. This is consistent with numerous previous studies and could not be simply explained by perceptual discriminability. Since we utilized a memory-guided saccade task, this indicates that the biased processing of the red color category does not rely on sustained sensory input and must therefore involve cortical areas associated with the highest levels of visual processing involved in visual working memory.

## Introduction

Color processing plays an important role in many oculomotor behaviors like pursuit eye movements (Tchernikov and Fallah, 2010), saccadic eye movements (Itti and Koch, 2000; McPeek and Keller, 2001; Ludwig and Gilchrist, 2003; Mulckhuyse et al., 2009), visual search (Green and Anderson, 1956; Treisman and Gelade, 1980; D'Zmura, 1991; Bauer et al., 1996a,b, 1998; Lindsey et al., 2010), and attentional selection (Folk et al., 1992, 1994; Wolfe and Horowitz, 2004; Pomerleau et al., 2014). Examinations of these behaviors have provided evidence that the color representations that influence oculomotor planning are encoded in multi-dimensional color space similar to how perceived color is encoded. For example, it has been demonstrated that the speed of pursuit eye movements to one of two differently-colored, superimposed random dot kinematograms (RDKs) moving in opposite directions is linearly proportional to the distance between the colors in CIE (*x*,*y*) color space (Tchernikov and Fallah, 2010). Similarly, it has been demonstrated that the discriminability of a target from distractors in color visual search is determined by the linear discriminability of the target from the distractors in CIE (*x*,*y*) color space (D'Zmura, 1991; Bauer et al., 1996a,b, 1998; c.f. Lindsey et al., 2010). However, whether the color representations utilized for saccade planning are also encoded in a multi-dimensional color space remains unclear.

There is physiological (Bichot et al., 1996; Sato and Schall, 2003; Sato et al., 2003) and behavioral (McPeek and Keller, 2001; Ludwig and Gilchrist, 2003; Mulckhuyse et al., 2009) evidence that color, at least categorically, modulates saccade planning. Additionally, there is preliminary evidence to suggest that the oculomotor system encodes potential saccade goals in perceptual color space: First, White et al. (2009) demonstrated that neurons in the intermediate layers of the superior colliculus (SCi)—a critical neural substrate for the generation of saccades (Robinson, 1972; Wurtz and Goldberg, 1972) and oculomotor target selection processing (Basso and Wurtz, 1997, 1998; McPeek and Keller, 2002, 2004)—have inherent color sensitivities characterized by very broad tuning profiles in DKL color space. Second, the color representations in the oculomotor system almost certainly originate from cortex as the alternative retinotectal pathway is colorblind (Schiller et al., 1979) and the long onset latencies of color signals in SCi (~80–90 ms; White et al., 2009) are inconsistent with the characteristically short onset latencies of the retinotectal pathway (11–27 ms; Schiller and Malpeli, 1977). Furthermore, the latency differences between color and luminance signals in the oculomotor system observed either physiologically (White et al., 2009) or inferred from psychophysics (Kehoe and Fallah, 2017) are very similar to the latency differences observed between the cortical dorsal and ventral visual processing streams (Schmolesky et al., 1998). This suggests that color representations in the oculomotor system are processed through the ventral processing stream specifically, along which wavelength representations are transformed into perceived color representations in area V4 (Schein and Desimone, 1990; Conway and Livngstone, 2006; Conway et al., 2007). The ventral visual processing stream is also richly interconnected with the frontal eye field (FEF) (Felleman and Van Essen, 1991; Schall et al., 1995), another critical neural substrate for oculomotor target selection processing (Segraves and Goldberg, 1987; Dassonville et al., 1992; Schlag-Rey et al., 1992) that integrates complex visual representations and higher cognitive factors (Bichot et al., 1996; Moore and Fallah, 2001, 2004; Sato et al., 2003; Thompson et al., 2005; Kastner et al., 2007). Third, there is an extensive degree of overlap in the neural circuitry that subserves saccadic eye movements, pursuit eye movements, and visual search (Schiller and Tehovnik, 2001; Krauzlis, 2005; Awh et al., 2006), and previous experiments have demonstrated that pursuit eye movements (Tchernikov and Fallah, 2010) and visual search (D'Zmura, 1991; Bauer et al., 1996a,b, 1998) are influenced by the color space relationships between stimuli. These observations suggest that perceptual color space representations could mediate saccadic target selection processing; however, whether and how they do so warrant further investigation.

One informative method to examine the factors that mediate saccadic target selection is to examine saccade curvature elicited from remote distractors. Physiological studies have demonstrated that saccade curvature is elicited from unresolved competition between potential saccade goals in the epoch immediately prior (~30 ms) to the initiation of saccades (McPeek et al., 2003; McPeek, 2006). Saccade curvature toward a distractor reflects distractor-related excitation (McPeek et al., 2003; McPeek, 2006), while curvature away from a distractor likely reflects distractor-related inhibition (Aizawa and Wurtz, 1998). Critically, the magnitude of unresolved activity encoding a distractor vector in this epoch is proportional to the magnitude of saccade curvature for saccades curved both toward (McPeek et al., 2003; Port and Wurtz, 2003; McPeek, 2006) and away from a distractor (White et al., 2012). Therefore, saccade curvature provides an index of the inherent competitiveness of a non-target stimulus during target selection processing. In fact, behavioral studies have demonstrated that saccade curvatures are greater when the color of a task irrelevant, peripheral distractor is congruent with a saccade target than when it is incongruent (Ludwig and Gilchrist, 2003; Mulckhuyse et al., 2009). However, these studies do not provide insight into whether potential saccade goals are encoded in perceptual color space, as with pursuit (Tchernikov and Fallah, 2010). This could be examined by measuring the magnitude of saccade curvature as a continuous function of the distance between a cued saccade target and a peripheral distractor in perceptual color space. Interestingly, under such experimental conditions, previous research suggests that two possible phenomena may arise: (1) feature-based surround suppression and (2) hierarchical color selection.

Researchers have demonstrated that there is an inhibitory annulus surrounding the locus of attention giving the “beam” of attention a difference of Gaussians (DoG) or Mexican hat wavelet spatial profile (Slotnick et al., 2002; Müller and Kleinschmidt, 2004; Hopf et al., 2006). This so-called “surround suppression” was first predicted by Tsotsos (1990) and incorporated into the computational theory of visual attention “selective tuning” (ST) (Tsotsos et al., 1995; Tsotsos, 2011). Critically, however, ST predicts that surround suppression should also be observed in the feature domain of an attended feature, which has been confirmed for orientation (Tombu and Tsotsos, 2008; Loach et al., 2010), and more recently, for color (Störmer and Alvarez, 2014).

Störmer and Alvarez (2014) utilized a perceptual discrimination task in which human participants covertly monitored two differently colored RDKs located in opposite hemifields and discriminated whether coherent motion emerged in one of the RDKs. They observed that discrimination performance was highest when the colors of the two attended RDKs were close *or far* in color space and was lowest when colors were at an intermediate distance. This result is characteristic of attention-based surround suppression in the feature domain of the attended feature (i.e., color) as predicted by ST (Tsotsos et al., 1995; Tsotsos, 2011). We therefore predict that if attention is cued to a color saccade target and the inherent competitiveness of a color distractor during target selection processing is measured, saccade curvature should vary as a function of the distance between target and distractor in color space with a DoG profile.

Research examining the influence of color on oculomotor processing has provided evidence for a color hierarchy of attentional selection, first proposed by Tchernikov and Fallah (2010). They observed that when participants executed a saccade to an aperture containing two superimposed RDKs with equal luminance and velocity, but with different directions and colors, participants made smooth pursuit eye movements to one of the two colors in the absence of any task instructions to do so, demonstrating automatic selection. Critically, however, they also observed a color hierarchy of selection in which red was selected over green, yellow, and blue; there was a weak preference of green over yellow and blue; and blue was not preferred over other colors. Lindsey et al. (2010) then demonstrated that visual search for desaturated color targets imbedded amongst saturated and white distractors that were equidistant from the target in CIE (*x*,*y*,*Y*) color space was more efficient for “reddish” targets than “purplish” targets. These effects were independent of target luminance differences, the perceptual similarity between targets and distractors, linear discriminability of targets from distractors in CIE (*x*,*y*,*Y*) color space, and lexical color category membership. In addition to these behavioral results, Pomerleau et al. (2014) measured event-related potentials during a visual search task and observed that the N2pc subcomponent, which is indicative of the contralateral deployment of attention (Luck, 2014), had a shorter latency for red and blue than green and yellow targets. This result provided the first electrophysiological evidence for hierarchical differences in the attentional selection of color. Even more recently, Blizzard et al. (2017) demonstrated that red stop signals elicited faster response inhibition than green stop signals on a stop signal task, thus demonstrating hierarchical color selection involvement in higher stages of cognitive processing.

These results strongly suggest that reddish hues receive preferential or biased processing in the visual system compared to other colors. However, these few studies of the color hierarchy give conflicting accounts of the level in the visual processing hierarchy at which biased color selection occurs. For example, Lindsey et al. (2010) provided evidence that the varying proportions of color signals from the L, M, and S color channels in the earliest stages of color processing explain the selection bias, whereas the results of Blizzard et al. (2017) suggested that the biased selection of certain colors must take place at higher levels of cognitive processing associated with attentional deployment and executive function.

The purpose of the current research was to (1) investigate whether color representations are encoded in perceptual color space by the oculomotor system. This was examined with a memory-guided saccade task in which participants were instructed to saccade to the remembered location of a color target displayed amongst color distractors. We then measured saccade curvature as a function of the CIE (*x*,*y*) color space distance between the target and an isolated distractor to determine if saccade planning is systematically modulated by the perceptual color space relations between potential saccade goals. We utilized CIE (*x*,*y*) color space as it represents color encoding in cortical structures like V4 (Schein and Desimone, 1990) and is therefore more appropriate for studying the role of color in higher-order cognitive processes such as sustained memory representations than other color space conceptualizations representing lower-level color representations in, for example, lateral geniculate nucleus such as Derrington-Krauskopf-Lennie color space (Derrington et al., 1984). We analyzed saccade curvatures as they are proportional to distractor-related unresolved competition immediately prior to the initiation of saccades and are therefore indicative of the inherent competitiveness of a distractor (McPeek et al., 2003; Port and Wurtz, 2003; McPeek, 2006; White et al., 2012). Furthermore, saccade curvatures are modulated by color (McPeek and Keller, 2001; Ludwig and Gilchrist, 2003; Mulckhuyse et al., 2009) and are elicited by remembered stimuli (Belopolsky and Theeuwes, 2011; Belopolsky and Van der Stigchel, 2013). Saccade curvature direction (i.e., toward vs. away from distractors) is largely indicative of distractor processing time (McSorley et al., 2006, 2009; Kehoe and Fallah, 2017), which is likely due to the transition from excitatory to inhibitory oculomotor distractor-related processing over time (McPeek and Keller, 2002). As we were specifically interested in examining potential differences in the inherent competitiveness of color distractors in the current study, we analyzed absolute saccade curvature. If the color space distance between targets and distractors functionally modulates absolute saccade curvatures, this would provide evidence that the oculomotor system encodes color stimuli in perceptual color space. (2) We examined whether the competitiveness of distractors was modulated by surround suppression in the color feature-domain by examining whether inherent competition (i.e., saccade curvature) varied as a function of color space distance specifically with a Mexican hat or quadratic polynomial mathematical profile, as surround suppression is characterized by a DoG (Tsotsos et al., 1995; Tsotsos, 2011) and since a Mexican hat approximates a DoG as does a quadratic function within a limited range. (3) We investigated whether there was a hierarchical, biased selection of certain color categories (red > green > yellow > blue) for saccades as with pursuit eye movements (Tchernikov and Fallah, 2010) by examining overall color selection differences between color categories. We then examined whether this was accompanied by hierarchical differences in the strength and speed of saccadic vector encoding by examining whether there were any hierarchical differences in the inherent competitiveness (i.e., saccade curvature) of isolated distractors, error proportion, mean saccadic reaction time (SRT), and saccadic precision as a hierarchical function of color category. (4) We examined whether the biased selection of color stimuli that characterizes the color hierarchy occurs at low or high levels of color representation. We therefore utiliized a memory-guided saccade task. First, because it relies on sustained visual working memory representations in the anterior most areas of the frontoparietal network like dorsolateral prefrontal cortex (Goldman-Rakic, 1995) as supposed to sustained sensory input. Therefore any hierarchical color effects would indicate that color selection biases either occur or are maintained at the highest levels of visual processing. Second, target selection processing in the delayed saccade paradigm is highly similar to visually-guided target selection processing (Segraves and Goldberg, 1987; Schall, 2004) and our results can therefore be generalized to the broader oculomotor target selection literature.

## Methods

### Subjects

Thirty York University undergraduate students (17 to 48-year-olds, 9 males) participated in the experiment for course credit. Participants had normal or corrected-to-normal visual acuity and had normal color vision as assessed by Ishihara color plates (Ishihara, 2006). Written informed consent was obtained prior to participation in accordance with the Declaration of Helsinki. York University's Human Participants Review Committee approved all experimental procedures.

### Visual stimuli

**Figure 1:**
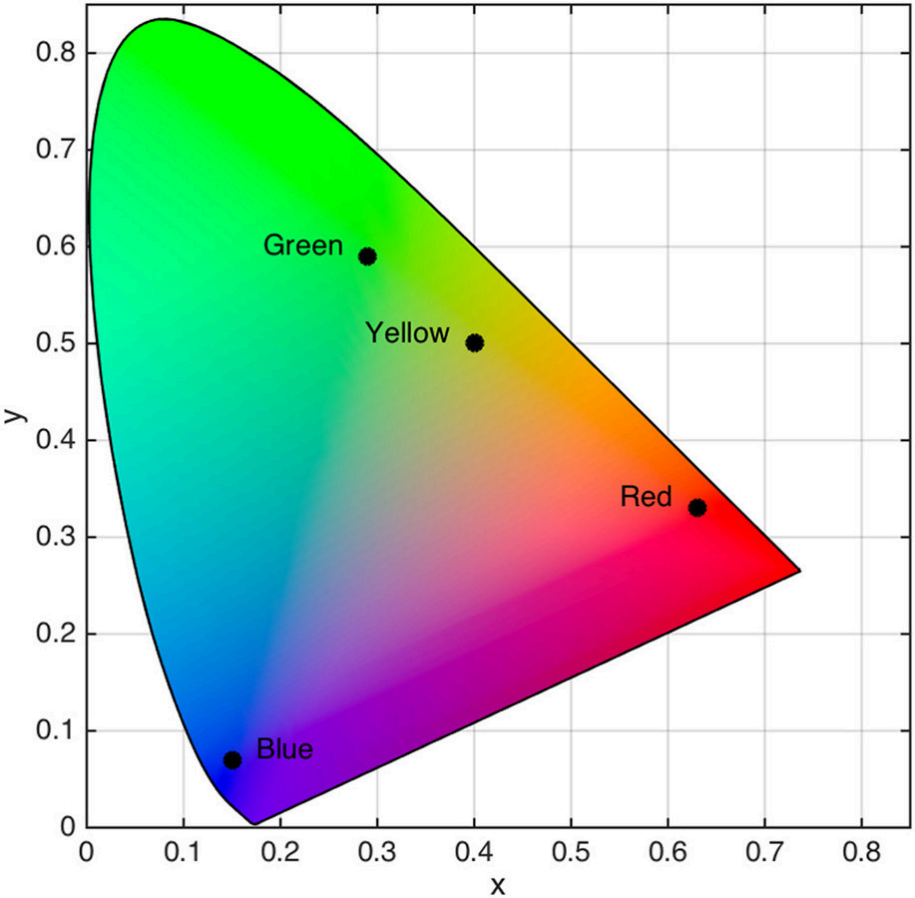
Locations of isoluminant target color categories in CIE (*x*,*y*) color space. Black dots with associated labels denote color category locations.

The saccade target was a red (*x* = 0.63, *y* = 0.33, *L* = 12.07 cd/m^2^), green (*x* = 0.29, *y* = 0.59, *L* = 12.00 cd/m^2^), yellow (*x* = 0.40, *y* = 0.50, *L* = 12.09 cd/m^2^), or blue (*x* = 0.15, *y* = 0.07, *L* = 12.04 cd/m^2^) square that subtended 2.0° × 2.0° (see Figure 1). Placeholders were gray (*x* = 0.28, *y* = 0.30, *L* = 11.20 cd/m^2^) squares that subtended 2.0° × 2.0°. Stimuli were embedded in a black (*x* = 0.26, *y* = 0.24, *L* = 0.22 cd/m^2^) background displayed on 21-inch CRT monitor (60 Hz, 1024 × 768). Color and luminance were calibrated using a spectrophotometer (PR-655, Photo Research, Syracuse, NY). Participants observed stimuli in a dimly lit room from a distance of 57 cm with a headrest used to stabilizing their head position.

### Apparatus

Stimulus presentation was controlled on a computer running Presentation software (Neurobehavioral Systems, Berkeley, CA) and a serial response box (Cedrus, San Pandro, CA). Eye position was sampled using an infrared eye tracker (500 Hz, Eyelink II, SR Research, Ontario, Canada). The camera was calibrated using a nine-point grid. Calibration was conducted at the beginning of each experimental run. Drift-corrections were conducted prior to each block and as needed.

### Procedure

**Figure 2:**
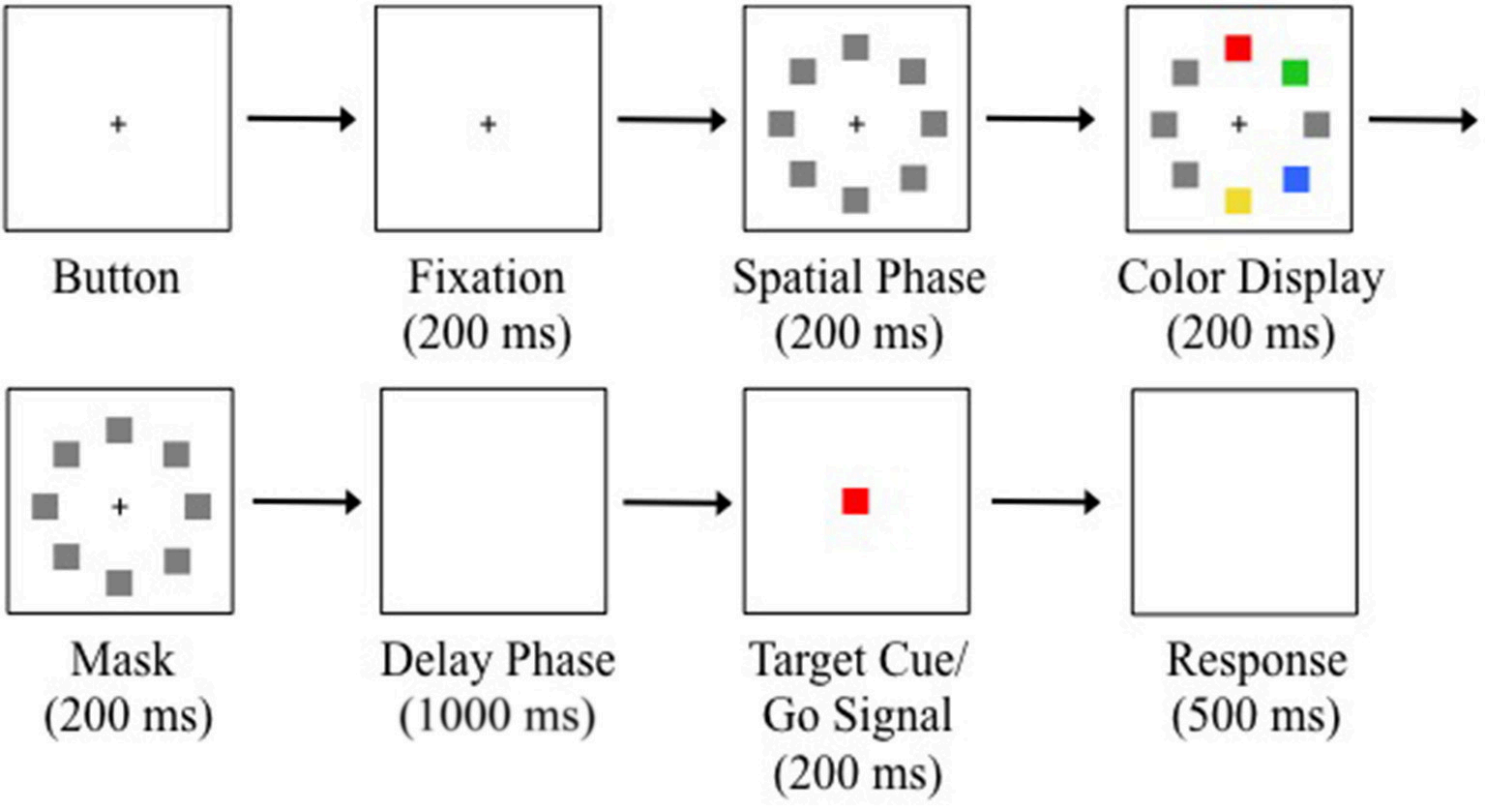
Example trial sequence with a red target. Trials were initiated after button pressing and fixating for 200 ms. Gray placeholders then occupied all the potential target positions for 200 ms. Four locations were randomly selected to display colored squares for 200 ms. The colored squares were masked for 200 ms. A blank display was presented for 1000 ms to produce memory-guided saccades. The central target cue appeared for 200 ms. Offset of the target cue was the saccadic go-signal. Participants were given 500 ms to execute a saccade. Note that there was an isolated distractor on this example trial (see Saccade Detection and Data Analysis).

Trials were initiated by a button press on the serial response box and then maintaining fixation (1.89° square window) on a white central fixation cross (0.4° × 0.4°) for 200 ms (see Figure 2). Eight placeholders centered to the circumference of an imagery circle (radius = 7.5°) at positions along the cardinal and oblique axes were displayed for 200 ms to indicate all potential target positions (spatial phase). A randomly sampled subset of four placeholders was then replaced with red, green, yellow, and blue squares and displayed for 200 ms (color display). The colored squares then reverted back to gray placeholders and were displayed for 200 ms (mask phase). The placeholders and fixation cross were removed from the display creating a blank, gray background displayed for 1000 ms (delay phase). A target color was randomly selected from the four colors and a target cue was displayed at central fixation for 200 ms. The offset of the central target cue served as the go-signal to execute a saccade to the remembered location of the target color from the previous color display. Participants were instructed to maintain fixation throughout the trial until presented with the go-signal. The trial was complete when a saccade was made to the location of the target colored square (correct) or a non-target colored square (incorrect) from the color display, or until 500 ms had elapsed since the go-signal (time out). An error tone signified a time-out or failure to maintain fixation. Trials with a timeout or fixation break were reinserted randomly back into the block. There were eight trials for each of the four target colors on every block. Participants completed 10 blocks of 32 trials for a total of 320 trials.

### Saccade detection and data analysis

Customized MATLAB (MathWorks, Natick, MA) algorithms were used to detect, visualize, filter, and analyze saccades. Trials that contained blinks, corrective saccades, saccade amplitudes < 1°, endpoint deviations > 6° from the center of the selected stimulus, or fixation drifts > 1° during the presaccadic latency period were excluded from further analysis.

To examine unresolved competition between stimuli during saccade planning, we analyzed two saccade curvature metrics: (1) *sum curvature*, the sum of all orthogonal deviations from a straight line between the start and endpoint of saccade trajectories sampled by the eye tracker; and (2) *max curvature*, the maximum orthogonal deviation from a straight line between the start and endpoint of saccade trajectories. To compute these metrics, we first translated the start point of each saccade to the Cartesian origin and then trigonometrically rotated each saccade so that the endpoint was aligned to the positive *y*-axis. Therefore, the saccade curvature metrics were signed so that a negative sign indicates deviation (i.e., curvature) in the counter-clockwise (CCW) direction, and conversely, a positive sign indicates deviation in the clockwise (CW) direction. This method was ideal for examining saccade curvature on circular displays with randomized target and distractor locations. Saccades with a sum curvature greater than three standard deviations from the mean were not analyzed for any curvature analyses.

For all saccade curvature analyses, we only analyzed a subset of correct trials with the following characterizations: (1) a single distractor occupied the stimulus positions 45° or 90° CCW to the target and there were no distractors occupying either of the stimulus positions 45° or 90° CW to the target (subsequently referred to as the CCW condition); (2) a single distractor occupied the stimulus positions 45° or 90° CW to the target and there were no distractors occupying either of the stimulus positions 45° or 90° CCW to the target (subsequently referred to as the CW condition); or (3) no distractors occupied any of the stimulus positions within 90° of the target in either the CCW or CW direction (subsequently referred to as baseline). We refer to trials in the CCW and CW conditions as isolated distractor trials. Unpredictable distractors located 135° from a target position reportedly elicit only a marginal effect on saccade curvature (McSorley et al., 2009) and were therefore not considered in the current analyses. First, we examined whether baseline curvature was significantly biased in a particular direction by comparing baseline curvature to zero. Next, to ensure that the isolated distractor 45° or 90° away from the target position elicited a systematic effect on saccade curvature, we compared saccade curvatures in the CCW and CW conditions relative to baseline (i.e., using unbiased curvatures) to determine if saccades curved away from the isolated distractors as is commonly observed in the remote distractor paradigm (Doyle and Walker, 2001; Tipper et al., 2001). We used unbiased curvature since a significant bias may uniformly shift curvatures in either the CCW or CW direction such that they do not actually curve in opposite directions (where the sign of the metrics indicates the direction) even though the distractors are located in opposite directions.

Next, to investigate whether potential saccade goals were encoded in CIE (*x*,*y*) perceptual color space, we analyzed whether unresolved competition between potential saccade goals (as indexed by saccade curvature) was modulated by the color space distances between the target and isolated distractors, referred to as *target-distractor color space distance*. This analysis was performed in several steps: First, we computed an exhaustive list of pairwise Euclidian distances between each color stimulus in CIE (*x*,*y*) color space (see Table 1) using the Pythagorean equation and categorized the color space distance between the target and the isolated distractor on every trial. Second, we computed mean saccade curvature as a function of target-distractor color space distance separately for the CCW and CW conditions. We then averaged together the absolute, unsigned saccade curvature from the CCW and CW conditions for each target-distractor color space distance, as we were ultimately interested in examining the average magnitude of saccade curvature (i.e., inherent competitiveness of distractors) as a function of target-distractor color space distance regardless of the relative position of the isolated distractor to the target and the direction of the curvature. Furthermore, averaging the CCW and CW conditions would compensate for any curvature bias and thus generalize the current results such that they are not limited to any particular spatial arrangement of a color distractor relative to the target. Third, we examined absolute mean saccade curvature as a function of target-distractor color distance with regression analyses. To determine whether the inherent competitiveness of isolated distractors was modulated by surround suppression in color space, we fit the data with a Mexican hat wavelet model (a close approximation to the DoG function):

$$\begin{array}{l} f\left(x|\alpha ,\sigma ,\kappa \right)=\left(\alpha -y_{n}\right)\cdot \left(1-\frac{x^{2}}{\kappa \sigma^{2}}\right)\cdot e^{-\frac{x^{2}}{2\sigma^{2}}}+y_{n}, \end{array}$$

where α is the function ceiling, σ is the width of the function, κ was included to scale the depth of the inhibitory annulus, *y*_*n*_ is the baseline of the function and was set to the mean saccade curvature (*y*_*i*_) observed at the furthest target-distractor color space distance (*n*), and minima are located at $x=\;\pm \sqrt{\kappa +2}\sigma$. Next, we fit the data with a generic quadratic polynomial model:

$$\begin{array}{l} f\left(x|a,b,c\right)=\;ax^{2}+bx+c. \end{array}$$

**Table 1:** Distances in CIE (*x*,*y*) color space between each successive pair of color stimuli and the average distance of each color stimulus from the remaining color stimuli.

**Pairwise CIE (*x*,*y*) Distances**					**Average Distance**
	**Red**	**Green**	**Yellow**	**Blue**	**(Discriminability)**
Red	–	0.43	0.29	0.55	0.42
Green	0.43	–	0.14	0.54	0.37
Yellow	0.29	0.14	–	0.50	0.31
Blue	0.55	0.54	0.50	–	0.53

*The average distance was utilized as an index of target color discriminability*.

Reducing the number of fitted free parameters in the Mexican hat model ensured an equal number of fitted parameters between models. Functions were fit using a custom implementation of the maximum likelihood estimation method by maximizing the following Gaussian log-likelihood function:

$$\begin{array}{l} l\left(\theta |y\right)=\;\overset{n}{\underset{i=1}{\sum }}log\left[\;\phi \left({ŷ}_{i}|y_{i},\sigma_{i}\right)\;\right], \end{array}$$

where θ is the vector of fitted parameters in either the Mexican hat wavelet [θ = (α, σ, κ)′] or quadratic [θ = (*a, b, c*)′] models, *n* is the number of target-distractor color space distances being fitted to the model, ϕ is the Gaussian normal probability density function, ŷ_*i*_ is the saccade curvature value predicted by the model with parameters θ for the *i*th target-distractor color space distance, *y*_*i*_ is the average saccade curvature value observed for the *i*th target-distractor color space distance, and σ_*i*_ is the standard deviation for the *i*th target-distractor color space distance. The goodness-of-fits were evaluated using an *F*-test. If both models provided a significant fit to the data, the best fitting model was determined by performing an *F*-test on the ratio of the sum of squared residuals from each fitted model with *n*−*k* degrees of freedom, where *k* is the number of fitted parameters (see Kehoe and Fallah, 2017). Furthermore, to provide further evidence of a functional relationship between saccade curvature and target-distractor color space distance, we analyzed the data with a quadratic planned contrast.

To examine whether there was an overall selection bias on color categories, we analyzed potential categorical color differences between *overall selection proportion*, which was the proportion of total trials (i.e., correct and incorrect) in which a particular color category was selected as a saccade target. This required us to include the error trials back into the data and then remove them for subsequent analyses.

We then analyzed whether the inherent competitiveness of distractors was hierarchically modulated by the color categories of the isolated distractors. As with target-distractor color space distance, absolute, unsigned saccade curvature was averaged between the CCW and CW conditions for each isolated distractor color. All subsequent analyses were performed on all correct trials of all trial types (i.e., isolated distractors and no isolated distractors).

Next, to examine whether the strength and speed of saccade target color encoding varied as a function of the target color category, we examined three metrics related to the strength and speed of target encoding: (1) *Proportion of errors*, which was the number of trials for a particular target color in which the saccade was executed to an incorrect location divided by the total number of trials with that target color. (2) *Saccadic reaction time* (SRT), which was the time between the go-signal and saccade initiation for correct saccades. (3) *Saccadic precision*, which was the area of a 95% confidence data ellipse fit to the 2-dimensional displacement between the target center and the saccade endpoints computed for each color and each participant for correct saccades (see Chen et al., 2011). Saccade precision was computed before and after translating the start-point of every saccade back to fixation. Our results demonstrated that this translation did not have a systematic effect on the results and thus we report the results for translated saccades.

Mean differences were analyzed with paired-samples *t*-tests or repeated-measures ANOVAs and Bonferroni *post-hocs*. If a Mauchley's test provided insufficient evidence for sphericity, the degrees of freedom of the ANOVA were adjusted using the Greenhouse-Geisser (ε ≤ 0.7) or Huynh-Feldt (ε > 0.7) adjustment. To examine whether there were hierarchical differences between color categories (red > green > yellow > blue) consistent with the color hierarchy (Tchernikov and Fallah, 2010), we examined planned polynomial contrasts of the color categories. As we performed parametric analyses, a Shapiro-Wilk test was used to determine whether the data were normally distributed. If this analysis provided insufficient evidence of normally distributed data (*p* < 0.05), the data was transformed to achieve normality using a log(*x* +1) transformation. If this did not successfully normalize the data, we alternatively utilized a $\sqrt[{3}]{x}$ transformation. These transformations were chosen because they are ideal for normalizing positively skewed data such as ours.

Lastly, for each categorical analysis discussed above, we performed a complimentary functional analysis to rule out perceptual discriminability as a possible explanation for any potential differences between color categories. First, the discriminability of each color was calculated by averaging the distance between one color category and all remaining color categories in CIE (*x*,*y*) color space (see Average Distance column in Table 1). Next, a linear regression analysis using ordinary least squares was performed to assess whether there was a functional relationship between the discriminability of each color category and the means utilized in the aforementioned categorical analyses.

## Results

*Chi*-squared goodness-of-fit tests were repeated for each participant to analyze the frequencies of correct and incorrect trials and ensure that individual participants correctly discriminated the target above chance on each experimental run (all *p*s < 0.05). As such, the average task performance of the group (*M* = 62.89%, *SE* = 2.71%) was significantly above chance level (25%), *t*_(29)_ = 13.96, *p* < 0.001, *d* = 2.55. Error trials were removed from the subsequent analyses with the exception of overall selection proportion. There was no difference across target color categories in the number of trials thrown out due to a large displacement (>6°) from the selected stimulus, χ${}_{\left(3,\text{N}=420\right)}^{2}$ = 0.59, *p* = 0.899 (see section Saccade Detection and Data Analysis).

### Color space encoding of isolated distractors

**Figure 3:**
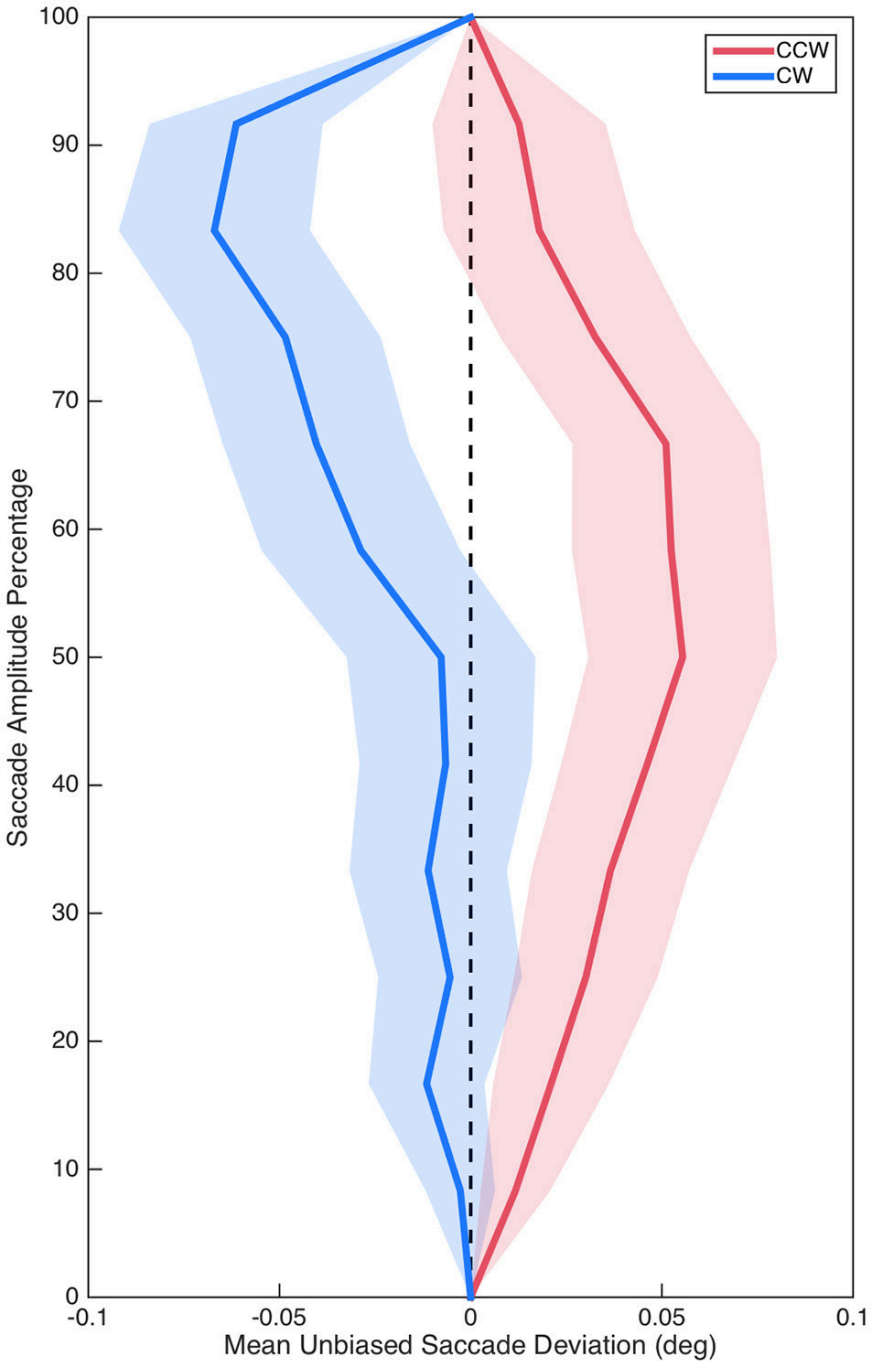
Mean unbiased saccade trajectories for saccades in the CCW (red) and CW (blue) conditions. Shading represents standard error.

Sum curvature was significantly biased in the CCW direction overall as demonstrated by comparing baseline (*M* = −0.80°, *SE* = 0.34°) to zero, *t*_(29)_ = 2.32, *p* = 0.027, *d* = 0.42. Critically, however, unbiased sum curvature was significantly different between the CCW (*M* = 0.74°, *SE* = 0.38°) and CW (*M* = −0.75°, *SE* = 0.43°) isolated distractor conditions, *t*_(29)_ = 2.87, *p* = 0.008, *d* = 0.52 (see Figure 3). Similarly, baseline max curvature (*M* = −0.05°, *SE* = 0.03°) was also biased in the CCW direction, *t*_(29)_ = 2.12, *p* = 0.042, *d* = 0.39; and unbiased max curvature was also significantly different between the CCW (*M* = 0.03°, *SE* = 0.03°) and CW (*M* = −0.07°, *SE* = 0.03°) isolated distractor conditions, *t*_(29)_ = 2.65, *p* = 0.013, *d* = 0.48. Saccade curvature differences between the CCW and CW isolated distractor conditions suggested that isolated distractors systematically modulated saccade curvature and thus validated examining saccade curvature on isolated distractors trials to investigate target selection competition between color stimuli.

**Figure 4:**
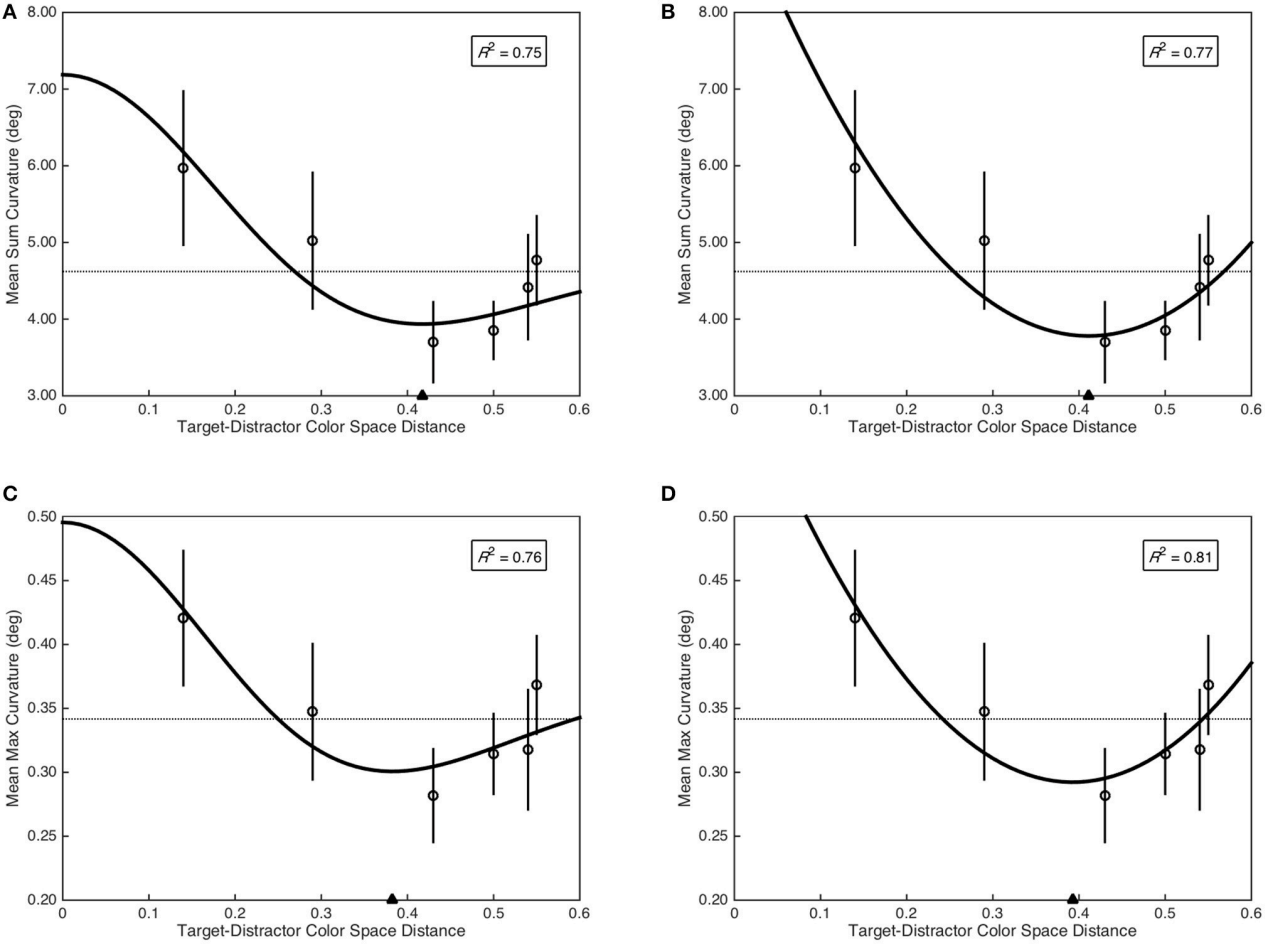
Saccade curvature as a function of target-distractor color space distance. Open circles represent mean saccade curvature and error bars represent standard error. Dashed horizontal lines indicate the grand mean across all color space distances. Filled triangle on the abscissa indicates the minima of the fitted models. The coefficient of determination is included in the top right of each plot. **(A)** Mean sum curvature fit as a function of the Mexican hat model. **(B)** Mean sum curvature fit as a function of the quadratic model. **(C)** Mean max curvature fit as a function of the Mexican hat model. **(D)** Mean max curvature fit as a function of the quadratic model.

The Mexican hat model provided a marginal fit of sum curvature as a function of target-distractor color space distance, *F*_(2, 3)_ = 6.25, *p* = 0.085, *R*^2^ = 0.75 (see Figure 4A); as did the quadratic model, *F*_(2, 3)_ = 7.44, *p* = 0.069, *R*^2^ = 0.77 (see Figure 4B). Critically, the minima of the fitted Mexican hat (*x*_min_ = 0.42) and quadratic (*x*_min_ = 0.41) models were nearly identical. To provide additional evidence for a functional relationship between sum curvature and target-distractor color space distance, sum curvature was transformed to achieve normality [*log*(*x* + 1)] and was analyzed with a quadratic planned contrast, which demonstrated a significant quadratic contrast, *F*_(1, 29)_ = 5.41, *p* = 0.027, $\eta_{p}^{2}$ = 0.16. In contrast, the Mexican hat model did not provide a significant fit of max curvature as a function of target-distractor color space distance, *F*_(2, 3)_ = 5.31, *p* = 0.103, *R*^2^ = 0.76 (see Figure 4C). However, the quadratic model provided a marginal fit to the data, *F*_(2, 3)_ = 7.44, *p* = 0.069, *R*^2^ = 0.81 (see Figure 4D). As with sum curvature, the minima of the fitted Mexican hat (*x*_min_ = 0.38) and quadratic (*x*_min_ = 0.39) models were nearly identical for the max curvature data. Again, to provide additional evidence for a functional relationship between max curvature and target-distractor color space distance, max curvature was transformed to achieve normality ($\sqrt[{3}]{x}$) and was analyzed with a quadratic planned contrast, which demonstrated a significant quadratic contrast, *F*_(1, 29)_ = 4.76, *p* = 0.037, $\eta_{p}^{2}$ = 0.14.

### Hierarchical differences between color categories

#### Color selection bias

**Figure 5:**
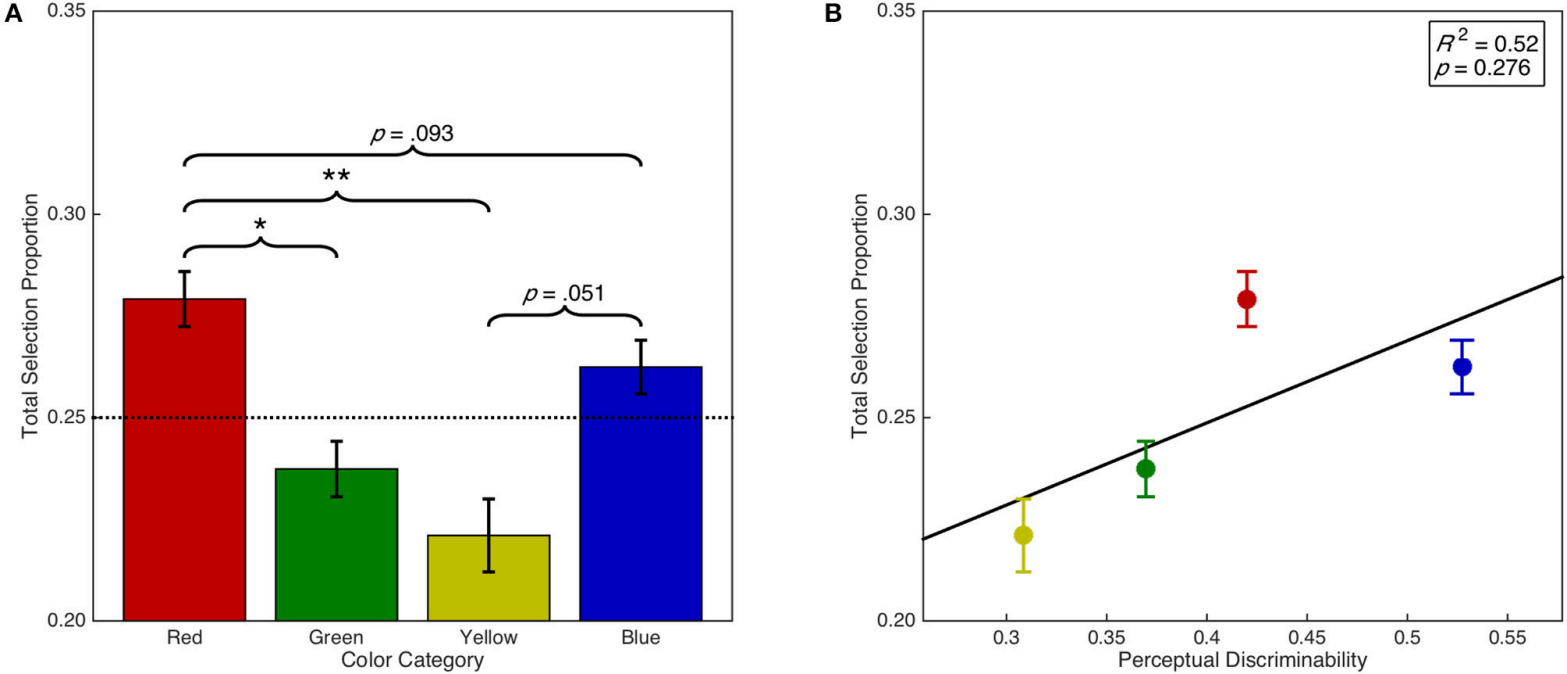
Selection bias as a function of color category and discriminability. Error bars represent standard error. Dashed horizontal line indicates chance. **(A)** Overall color selection proportion as a function of color category. **(B)** Overall color selection proportion as a function of color discriminability in CIE (*x*,*y*) color space. Panel includes line of best fit, the coefficient of determination, and significance level from the regression analysis. ^*^p < 0.05. ^**^p < 0.01.

Error trials were added back into the data in order to analyze the proportion of trials on which a particular color was selected regardless of the specified target color (i.e., regardless of task instructions) to investigate whether there was a bias for the selection of certain colors. We therefore calculated the proportion of total trials on which each color was selected, which is a combination of both the discrimination accuracy and the error selection bias for each color. This analysis demonstrated that there was a significant main effect of color category on overall selection proportion, *F*_(1.97, 57.08)_ = 9.27, *p* < 0.001, $\eta_{p}^{2}$ = 0.24 (see Figure 5A). *Post-hoc* analyses demonstrated that overall, red was selected more often than green (*p* = 0.012) and yellow (*p* = 0.002). There were no other significant differences (*p*s > 0.05). However, red was selected marginally more often than blue (*p* = 0.093) and blue was selected marginally more often than yellow (*p* = 0.051). Furthermore, there was a significant linear contrast, *F*_(0.66, 19.03)_ = 10.72, *p* = 0.008, $\eta_{p}^{2}$ = 0.27; and quadratic contrast, *F*_(0.66, 19.03)_ = 12.69, *p* = 0.005, $\eta_{p}^{2}$ = 0.30. We then examined whether color selection biases can be simply explained by discriminability differences in color space (see Figure 5B). A linear regression analysis found insufficient evidence of a linear relationship between the overall selection proportion and discriminability in CIE (*x*,*y*) color space, *F*_(1, 2)_ = 1.55, *p* = 0.340, *R*^2^ = 0.52. This result suggests that the strong bias for red stimulus selection is independent of discriminability in color space.

#### Saccade curvature on isolated distractor trials

**Figure 6:**
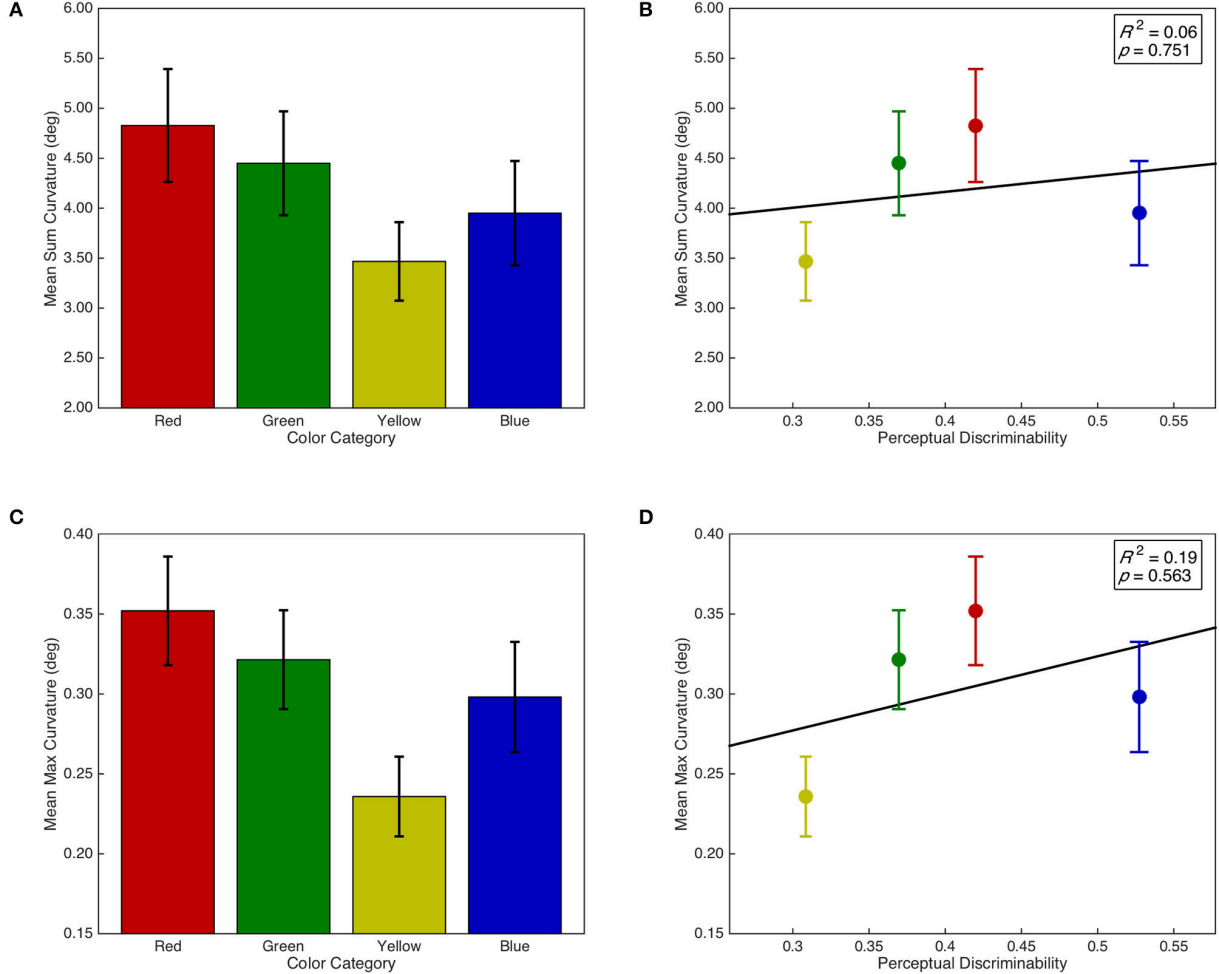
Saccade curvature as a function of isolated distractor category and discriminability. Error bars represent standard error. Right panels include line of best fit, the coefficient of determination, and significance level from the regression analyses. **(A)** Sum curvature as a function of isolated distractor color category. **(B)** Sum curvature as a function of isolated distractor discriminability in CIE (*x*,*y*) color space. **(C)** Max curvature as a function of isolated distractor color category. **(D)** Max curvature as a function of isolated distractor discriminability in CIE (*x*,*y*) color space.

Sum and max curvature were both normalized with a *log*(*x* + 1) transformation. There was no main effect of isolated distractor color category on sum curvature, *F*_(3, 87)_ = 1.85, *p* = 0.144, $\eta_{p}^{2}$ = 0.06; however, there was a marginal linear contrast, *F*_(1, 29)_ = 3.41, *p* = 0.075, $\eta_{p}^{2}$ = 0.11 (see Figure 6A). Furthermore, sum curvature was unrelated to the discriminability of isolated distractors in color space, *F* < 1, *R*^2^ = 0.06 (see Figure 6B). Similarly, there was a marginal main effect of isolated distractor color category on max curvature, *F*_(3, 87)_ = 2.62, *p* = 0.056, $\eta_{p}^{2}$ = 0.08; and there was a marginal linear contrast, *F*_(1, 29)_ = 3.65, *p* = 0.066, $\eta_{p}^{2}$ = 0.11 (see Figure 6C). Max curvature was also unrelated to the discriminability of isolated distractors in color space, *F* < 1, *R*^2^ = 0.19 (see Figure 6D).

#### Strength of saccade vector encoding per target color category

**Figure 7:**
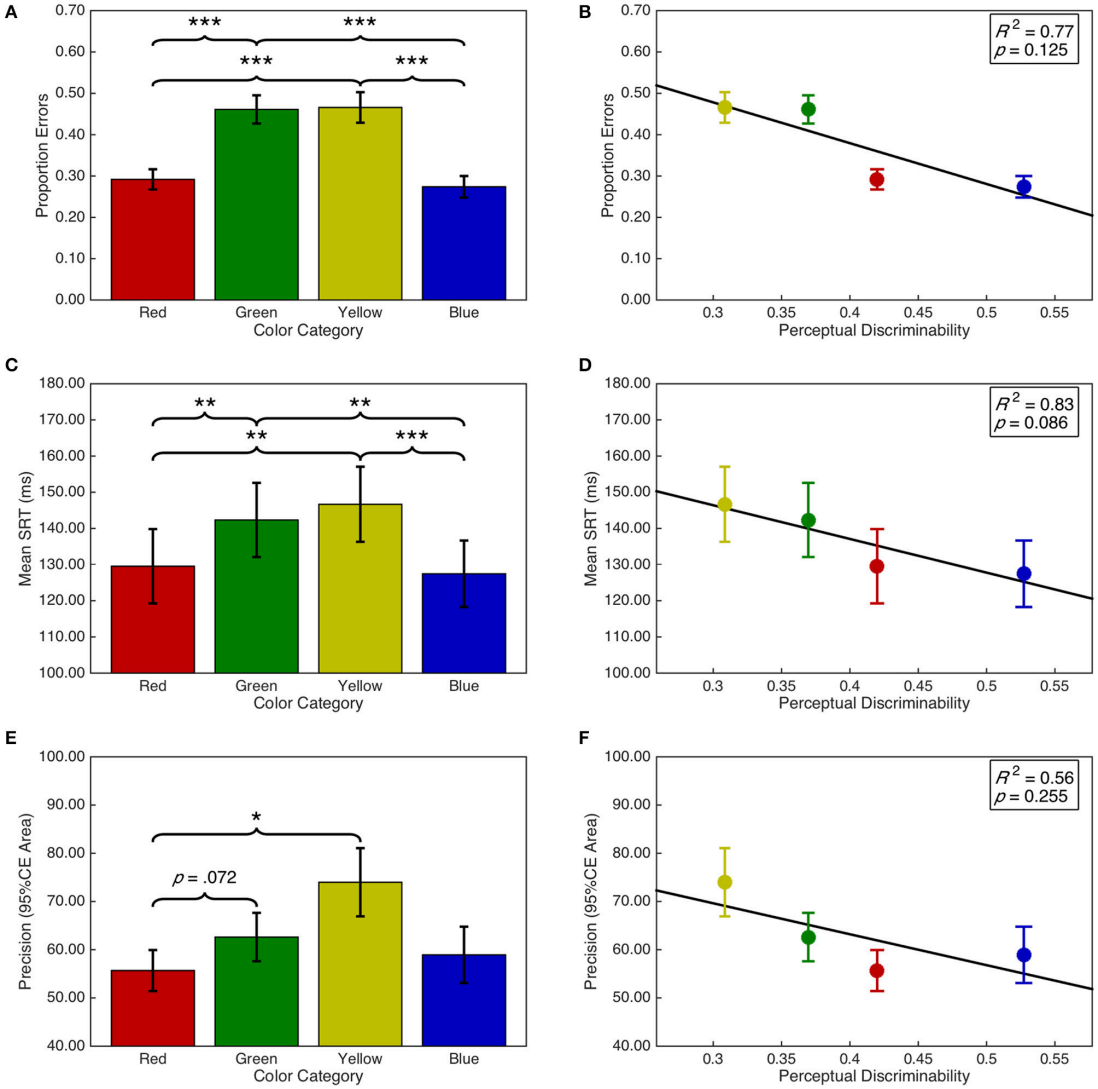
Task performance as a function of target color category and discriminability. Error bars represent standard error. Right panels include line of best fit, the coefficient of determination, and significance level from the regression analyses. **(A)** Proportion of errors per target color category. **(B)** Proportion of errors as a function of discriminability. **(C)** Mean SRT per target color category. **(D)** Mean SRT as a function of discriminability. **(E)** Mean precision per target color category. **(F)** Mean precision as a function of discriminability. ^*^p < 0.05; ^**^p < 0.001; ^***^p < 0.001.

There was a significant main effect of target color on the proportion of errors, *F*_(2.05, 59.52)_ = 37.11, *p* < 0.001, $\eta_{p}^{2}$ = 0.56 (see Figure 7A). *Post-hoc* analyses demonstrated that there were fewer errors for red targets than green (*p* < 0.001) and yellow (*p* < 0.001) targets. Similarly, there were fewer errors for blue than green (*p* < 0.001) and yellow (*p* < 0.001) targets. There were no other significant differences (*p*s > 0.05). Additionally, there was a significant quadratic contrast, *F*_(0.68, 19.84)_ = 59.70, *p* < 0.001, $\eta_{p}^{2}$ = 0.67; but insufficient evidence for a linear contrast, *F*_(0.68, 19.84)_ = 1.86, *p* = 0.263, $\eta_{p}^{2}$ = 0.04. These categorical differences were not explained by a linear relationship between target color space discriminability and proportion of errors, *F*_(1, 2)_ = 6.53, *p* = 0.125, *R*^2^ = 0.77 (see Figure 7B).

There was a significant main effect of target color category on saccadic reaction time, *F*_(3, 87)_ = 12.05, *p* < 0.001, $\eta_{p}^{2}$ = 0.29 (see Figure 7C). *Post-hoc* analyses demonstrated that SRTs were shorter for blue targets than for green (*p* = 0.001) and yellow (*p* < 0.001) targets. SRTs for red targets were shorter than for green (*p* = 0.004) and yellow (*p* = 0.003) targets. There were no other significant differences (*p*s > 0.05). As with proportion errors, there was a significant quadratic contrast, *F*_(1, 29)_ = 31.30, *p* < 0.001, $\eta_{p}^{2}$ = 0.52; but insufficient evidence for a linear contrast, *F* < 1. This was accompanied by a marginal, negative linear relationship between discriminability and saccadic reaction time, *F*_(1, 2)_ = 10.11, *p* = 0.086, *R*^2^ = 0.83 (see Figure 7D). The regression line (ŷ = −93.21·*x*+174.38) indicates that SRTs decreased by 9.32 ms as the average distance between a target color and the remaining color distractors increased by 0.1 units in CIE (*x*,*y*) color space.

Saccade precision was transformed using a *log*(*x* + 1) transformation. There was a significant main effect of target color on saccade precision, *F*_(1.77, 51.21)_ = 5.96, *p* = 0.006, $\eta_{p}^{2}$ = 0.17 (see Figure 7E). *Post-hoc* analyses indicated that saccades were more precise for red targets than yellow (*p* = 0.022) targets and marginally more precise than for green targets (*p* = 0.070). There were no other significant differences (*p*s > 0.05). Furthermore, there was a significant quadratic contrast, *F*_(0.59, 17.07)_ = 8.57, *p* = 0.007, $\eta_{p}^{2}$ = 0.23; but insufficient evidence for a linear contrast, *F*_(0.59, 17.07)_ = 2.56, *p* = 0.132, $\eta_{p}^{2}$ = 0.08. There was insufficient evidence for a linear relationship between discriminability and saccade precision, *F*_(1, 2)_ = 2.50, *p* = 0.255, *R*^2^ = 0.56 (see Figure 7F).

## Discussion

A memory-guided saccade task with cued color targets and distractors was used to examine the role of color in saccadic target selection processing. We investigated whether color saccade targets are encoded in perceptual color space by the oculomotor system and whether cuing a target color would elicit surround suppression in oculomotor color space. Next, we examined whether there are hierarchical color differences in the selection and encoding of saccade target vectors to color stimuli, as has been observed with the biased automatic selection of colors for pursuit eye movements (Tchernikov and Fallah, 2010), and whether such hierarchical effects can be accounted for simply by differences in perceptual discriminability. This provided insight into the level in the visual processing hierarchy at which the biased selection of certain colors occurs.

### Behavioral relevance and featural encoding in the oculomotor system

In our first analysis, we examined whether the color representations utilized by the oculomotor system for saccade planning were encoded in perceptual color space by analyzing trials on which a correct saccade was made to the target and an isolated, peripheral distractor was within 90° of the target. On these isolated distractor trials, we examined saccade curvature elicited by the distractor as saccade curvature is indicative of the competition elicited by a competing saccade goal during target selection processing. This allowed us to determine whether distractor-related competition covaried as a function of the perceptual color space relations between potential saccade goals on the task. We utilized a preliminary analysis to determine whether isolated distractors had a modulatory influence on saccade curvatures. Therefore, we compared saccade curvature between trials on which the distractor appeared counterclockwise (CCW) to the target to trials on which the distractor appeared clockwise (CW) to the target, as previous experiments suggest that saccades should curve in opposite directions in these two contexts (Doyle and Walker, 2001; Tipper et al., 2001). Furthermore, we also calculated the curvature observed on trials with no isolated distractor to establish a baseline level of inherent saccade curvature, which is expected as saccades as idiosyncratically curved (Bahill and Stark, 1975) and previous studies of saccade curvature report directional biases (Doyle and Walker, 2001; McSorley et al., 2004). As such, we observed a significant curvature bias in the CCW direction. Therefore, to determine if saccades curved in opposite directions in the CCW and CW conditions, we examined the difference in unbiased saccade curvature (i.e., baseline subtracted) between these conditions. This analysis was ideal as it merely introduces a scalar offset between the means, one that makes any potential mean difference more interpretable, but does not change the outcome of any statistical analysis since the variances are unchanged. This analysis demonstrated that saccades curved in opposite directions in the CCW and CW conditions relative to the baseline, thus validating our use of saccade curvatures to examine differences in color encoding by the oculomotor system. As such, we then measured saccade curvature as a function of the CIE (*x*,*y*) color space distance between the target and distractor. Our data demonstrated that saccade curvature elicited by these isolated distractors was functionally related to the color space distance between the target and distractor in CIE (*x*,*y*) color space. This suggested that the color representations that are utilized by the oculomotor system to plan saccadic eye movements are encoded in perceptual color space as with pursuit eye movements (Tchernikov and Fallah, 2010) and visual search (D'Zmura, 1991; Bauer et al., 1996a,b, 1998).

Previous studies have found that remote distractor-related saccade curvature is greater when the featural similarity of the distractor to the target is categorically greater (Ludwig and Gilchrist, 2003; Mulckhuyse et al., 2009). However, the current results demonstrate that oculomotor suppression of a distractor can vary continuously along a particular feature dimension that determines the behavioral relevance of the distractor. This continuous relationship between saccade curvature and distractor behavioral relevance may result from a gradient of oculomotor activation/inhibition in critical oculomotor neural substrates such as SCi where the spatiotemporal interactions of multiple saccade vectors can elicit saccade curvature toward a distractor when the target and distractor vectors are co-activated (McPeek et al., 2003) or away from a distractor when the distractor vector is inhibited (Aizawa and Wurtz, 1998). This reasoning is supported by the observation that saccade curvature toward (McPeek et al., 2003) and away (White et al., 2012) from a distractor is correlated with the magnitude of neural activation encoding the distractor vector. Interestingly, in the critical oculomotor substrates that encode movement vectors and have been most strongly associated with saccade curvature, namely SCi (McPeek et al., 2003; Port and Wurtz, 2003; White et al., 2012) and FEF (McPeek, 2006), there are many cells with visuomotor properties and some that are strictly visual (SCi: McPeek and Keller, 2002; FEF: Mohler et al., 1973; Bruce and Goldberg, 1985). Therefore, what is not clear from the current experiment is whether the featural computations necessary to determine the behavioral relevance of the stimuli is performed locally in these oculomotor substrates or whether alternatively these areas integrate visual and cognitive signals to related saccade vectors and select a winner for the movement. The latter mechanism is supported by research demonstrating behavioral relevance or *priority* encoding in SCi (reviewed by Fecteau and Munoz, 2006) and with a winner-take-all saccade triggering mechanism in SCi (see White and Munoz, 2011 for a discussion). Critically, this latter mechanism is also consistent with the current observation of surround suppression.

### Surround suppression of oculomotor representations

The relationship between saccade curvature and target-distractor color space distance was marginally fit by both Mexican hat and quadratic polynomial functions, and when treating color space distances categorically, there was a quadratic contrast between the means. This is consistent with surround suppression of the cued saccade target color in color space and provides experimental support for selective tuning (Tsotsos et al., 1995; Tsotsos, 2011). Critically, if surround suppression modulates the strength of visuomotor representations in the oculomotor system, this implies that the featural computation necessary to determine the behavioral relevance of potential saccade goals was not performed locally in oculomotor substrates. This is because surround suppression in feature space results from task-based priming of a relevant visual feature at the top level of representation in the visual hierarchy and the subsequent recursive pruning of connections to unrelated features in a recurrent feedback sweep through the visual hierarchy (Tsotsos et al., 1995; Tsotsos, 2011). This theoretical prediction of ST has been well-supported by recent behavioral (Boehler et al., 2009; Hopf et al., 2010) and neurophysiological (Mehta et al., 2000; Roelfsema et al., 2007; Buffalo et al., 2010) experiments. Since the oculomotor system does not have intrinsic featural representations, this feedback sweep would not propagate through the oculomotor system. Therefore, surround suppression modulating oculomotor representation implies that sensory signals projected into the oculomotor system have already been attenuated in the respective representational networks from which they originate. The behavioral relevance of a potential oculomotor movement goal is likely then determined by the cumulative strength of the attenuated sensory signals across feature domains. Furthermore, we would not expect similar results had we pre-cued the memory-guided saccade target, as this would have introduced the influence of spatial attention, which over the considerably lengthy delay period of 1000 ms, likely would have eliminated the featural competition elicited by distractors that we observed.

### Attentional color hierarchy

We examined whether hierarchical differences between color categories were apparent in various aspects of saccadic target selection processing and saccadic vector encoding as they are in the task irrelevant selection of certain color categories for pursuit eye movements (Tchernikov and Fallah, 2010) or the task relevant selection of visual search targets (Lindsey et al., 2010; Pomerleau et al., 2014) and response inhibition signals (Blizzard et al., 2017). We began by examining whether there was an overall selection bias for certain color categories regardless of task instructions by adding error trials back into the dataset and examining the proportion of total trials on which any particular color category was selected. Consistent with the automatic (i.e., task irrelevant) color selection bias observed by Tchernikov and Fallah (2010) in which participants hierarchically selected color RDKs (red > green > yellow > blue) for pursuit despite never being instructed to do so, we observed a strong overall bias for selecting the color red over green and yellow, and marginally over blue, which was accompanied by a significant linear contrast between the aforementioned color categories. Next, we examined if the inherent competitiveness of an isolated distractor was also modulated by the color category of the distractor. Here we observed only a marginal hierarchical effect of color category, but it was qualitatively the same as the overall selection bias above. Neither the overall selection bias nor the marginal modulation of inherent competitiveness by color category could be explained by differences in the discriminability of color categories in CIE (*x*,*y*) color space.

The results from examining overall selection proportion and inherent competitiveness of isolated distractors were qualitatively distinct from those obtained by examining the speed and strength of saccadic vector encoding of the target color (i.e., SRT and the proportion of error trials respectively). Our data demonstrated that when saccades were cued to red targets, they were faster, more precise, and were executed with fewer errors than saccades directed to green and yellow targets. However, blue demonstrated a similar advantage for the proportion of errors and speed, but not for precision. These results were accompanied by significant quadratic contrasts between the aforementioned color categories for the proportion of errors, saccadic latency, and saccadic precision and with insufficient evidence for a linear contrast across these metrics. Clearly, blue was processed more efficiently or elicited more selection bias than would otherwise be predicted by the original color hierarchy (red > green > yellow > blue) of Tchernikov and Fallah (2010). Recall that overall selection proportion and distractor inherent competitiveness showed no such equal advantage for the blue color category. There are several possible explanations for this discrepancy.

Our data may suggest a fundamental variation on the color hierarchy specifically for the speed and strength of saccade vector encoding (red = blue > green = yellow). For example, faster saccades executed to both red and blue targets is consistent with Pomerleau et al. (2014) who observed faster N2pc ERPs to both red and blue targets than green and yellow targets in visual search. Such results could reflect a fundamental difference in the speed of transmission between red/blue and green/yellow color signals. However, this is unlikely given that red/blue and green/yellow color signals are projected through separate anatomical color channels (De Valois and De Valois, 1993). More plausible is that the proportion of errors and response latency metrics are reflective of the perceptual decision component of the task, which is highly related to the discriminability of the stimuli: a perceptual decision threshold is surpassed faster as the target becomes more easily perceptually discriminable (Hanes and Schall, 1996; Gold and Shadlen, 2000; Ditterich et al., 2003). We find this explanation more plausible given that subsequent analyses suggested that lower error rates and faster latencies for red and blue saccades might be explained by the perceptual discriminability of these colors in color space, as SRTs showed a marginal linear relationship with the discriminability of the current color categories in color space, and although there was an insignificant relationship for error rates, a subjective inspection of these means suggests that errors might vary as a sigmoidal or step-like function of discriminability in color space. These results were also consistent with previous studies demonstrating that the discriminability of color stimuli in color space facilitates the response latency in visual search (D'Zmura, 1991; Bauer et al., 1996a,b, 1998). Furthermore, higher perceptual discriminability between stimuli also improves the signal-to-noise ratio in the perceptual accumulation process (Gold et al., 1999; Romo et al., 2003), which could account for our observation of fewer errors for red and blue than green and yellow color categories.

The differences may also be explained by the fact that color was strictly task irrelevant in Tchernikov and Fallah and was crucial for the current task. As such, the perceptual discriminability of the stimuli may impede the featural processing of the saccade targets therefore degrading task performance as is observed in visual search (Verghese and Nakayama, 1994; Verghese, 2001). Another possibility is that the current effects were influenced by differences in the strength of visual working memory representations between color categories. Previous studies that have reported preferential processing for red over other color categories have utilized tasks in which color processing occurs during sustained color stimulation (Lindsey et al., 2010; Tchernikov and Fallah, 2010; Pomerleau et al., 2014; Blizzard et al., 2017). Given that the signal-to-noise ratio of prefrontal neural activity is proportional to visual working task performance (Sawaguchi and Goldman-Rakic, 1991, 1994; Williams and Goldman-Rakic, 1995), utilizing a task that relies on visual working memory color representations may have introduced the influence of perceptual discriminability on localization performance as decreased perceptual discriminability may have degraded working memory representations. In any case, our data provided evidence for privileged processing of red stimuli, consistent with a growing number of studies (Lindsey et al., 2010; Tchernikov and Fallah, 2010; Pomerleau et al., 2014; Blizzard et al., 2017). Therefore, the current results cannot be simply accounted for just by differences in perceptual discriminability and likely arise from an interaction of inherent selection biases for red and the perceptual discriminability of the color stimuli.

### Representational level of the selection bias

We were interested in determining where in the visual processing hierarchy does the biased selection and processing of color categories occur. We utilized a memory-guided saccade task and observed clear hierarchical effects in the selection and inherent competitiveness of color categories independent of perceptual discriminability. Given the delay period of 1000 ms, previous research suggests that the color representations that guided this saccade task were those maintained through recurrent projections in the cortical visual system (Lee et al., 2005) perhaps as early as V1 (Supèr et al., 2001), as supposed to feedforward sensory signals from the retina, which would have decayed after this considerable delay. Therefore, the current categorical color effects likely do not arise from differing proportions of chromatic photoreceptors as has been proposed for speeded visual search (Lindsey et al., 2010).

## Conclusion

We utilized a memory-guided saccade task with color targets and distractors to gain insight into various aspects of how color is encoded and selected for saccades by the oculomotor system. Our results demonstrated that there is a functional relationship between saccade curvature elicited by an isolated distractor and the CIE (*x*,*y*) color space distance between the target and the distractor suggesting that the oculomotor system encodes color in perceptual color space as with the visual system. Furthermore, cueing attention to a particular color elicited surround suppression in oculomotor color space as the functional relationship between saccade curvature and target-distractor color space distance was characterized by functions that approximate a DoG, which demonstrates attentional facilitation near an attended feature in feature space and suppression at an intermediate distance in feature space. These results suggested that oculomotor behavioral relevance is computed by integrating sensory and cognitive signals that have been attenuated based on task parameters. Our data also suggested that the visual system has an inherent bias for the selection and encoding of the color category red over other categories independent of perceptual discriminability, but the speed and accuracy of responding on a memory-guided saccade task is more related to the perceptual discriminability of the target stimulus relative to distractors. Finally, this experiment suggests that the color hierarchy arises from selection and encoding biases in the later stages of visual processing independent of feedforward sensory input.

## Author contributions

Research was designed by MF and implemented by DK. Data acquisition was conducted by MR. Data analysis was conducted by DK and MR. Data was interpreted by MF and DK. Manuscript was drafted by DK and MR. Final draft of manuscript was approved by MF.

### Conflict of interest statement

The authors declare that the research was conducted in the absence of any commercial or financial relationships that could be construed as a potential conflict of interest.
